# Supplementary material for: Identification of Genetic Variants Associated with Severe Myocardial Bridging through Whole-Exome Sequencing
Source: J Pers Med. 2023 Oct 18;13(10):1509. doi: 10.3390/jpm13101509 (PMC10608235; doi:10.3390/jpm13101509)
Supplement: Supplementary file 1 [file jpm-13-01509-s001.zip › Supplement S1_Baseline Characteristics of Subjects with Severe Myocardial Bridging or Normal Controls.pdf]

Supplement S1. Baseline Characteristics of Subjects with Severe Myocardial Bridging or Normal Controls

**Sample**	**Gender**	**Age (years)**	**Bridge length (mm)**	**Residual diameter during systole (%)**	**Luminal area reduction (%)**
Patient 1 with SMB	M	43	22.2	39.4	84.5
Patient 2 with SMB	F	43	21.6	28.3	92.0
Patient 3 with SMB	F	59	9.5	36.0	87.0
Patient 4 with SMB	M	50	40.0	36.7	86.5
Patient 5 with SMB	F	53	11.8	33.2	89.0
Patient 6 with SMB	M	41	12.4	34.9	87.8
Patient 7 with SMB	F	64	14.8	22.4	94.0
Patient 8 with SMB	M	44	22.3	17.9	96.8

Normal control 1	M	42	-	-	-
Normal control 2	F	44	-	-	-
Normal control 3	F	52	-	-	-
Normal control 4	M	50	-	-	-
Normal control 5	F	51	-	-	-
Normal control 6	M	42	-	-	-
Normal control 7	F	60	-	-	-
Normal control 8	M	38	-	-	-

Normal control subjects do not have any myocardial bridging or any type of coronary artery disease. SMB, severe myocardial bridging; Subj., subject; M, male; F, female; MB, myocardial bridging.
