# Supplementary material for: Identification of Genetic Variants Associated with Severe Myocardial Bridging through Whole-Exome Sequencing
Source: J Pers Med. 2023 Oct 18;13(10):1509. doi: 10.3390/jpm13101509 (PMC10608235; doi:10.3390/jpm13101509)
Supplement: Supplementary file 1 [file jpm-13-01509-s001.zip › Supplement S2_Rare Variants Potentially Pathogenic for Severe Myocardial Bridging.pdf]

Supplement S2. Rare Variants Potentially Pathogenic for Severe Myocardial Bridging

Chr	Position	Ref	Alt	Gene	Type	rs number	ClinVar	CADD	REVEL	TWB AF
1	236749160	C	T	ACTN2	ns SNV	rs573836993	CIP	14.08	0.097	N/A
1	240493438	G	A	GREM2	ns SNV	rs373941682	Pathogenic	22.9	0.031	0.002
1	160041491	G	A	KCNJ10	ns SNV	rs137853074	CIP	23.6	0.522	0.0035
1	77926863	C	T	NEXN	ns SNV	rs146245480	CIP	32	0.211	0.0075
1	26913775	AGGTGCGATG	-	NR0B2	frameshift deletion	rs540387719	Likely_pathogenic	N/A	N/A	N/A
1	156868588	C	T	NTRK1	ns SNV	rs202030811	CIP	33	0.089	0.0045
1	226882007	G	A	PSEN2	ns SNV	rs200636353	CIP	18.56	0.331	0.0055
1	237614742	G	A	RYR2	ns SNV	rs761770946	CIP	10.76	0.181	0.005
1	202963091	C	A	CYB5R1	ns SNV	rs368686887	N/A	35	0.86	0.0015
1	54190767	C	T	CYB5RL	ns SNV	rs191854671	N/A	32	0.762	0.0015
1	20652503	G	A	DDOST	ns SNV	rs530990753	N/A	33	0.837	0.001
1	8863299	T	A	ENO1	ns SNV	N/A	N/A	34	0.806	N/A
1	108929794	C	T	GPSM2	ns SNV	rs189033496	U/S	35	0.842	0.003
1	161120221	G	C	NIT1	ns SNV	rs201971149	N/A	26.1	0.818	0.002
1	233200177	T	C	PCNX2	ns SNV	rs775218559	N/A	29.1	0.775	N/A
1	226888902	G	T	PSEN2	ns SNV	rs574125890	U/S	25.4	0.945	0.006
1	26066382	A	G	TRIM63	ns SNV	rs758754060	N/A	32	0.894	N/A
2	237388032	C	T	COL6A3	ns SNV	rs115729513	CIP	20.2	0.178	0.0035
2	178548927	T	C	TTN	ns SNV	rs186234393	CIP	13.78	0.496	0.0035
2	178561041	C	T	TTN	ns SNV	rs376283153	CIP	18.93	0.14	N/A
2	178565578	G	A	TTN	ns SNV	rs185887755	CIP	20.8	0.533	0.0065
2	98121411	G	T	VWA3B	stopgain	rs139296152	CIP	25.4	N/A	0.0055
2	31381723	C	T	XDH	ns SNV	rs192453952	CIP	13.23	0.025	0.0075
2	168944720	C	T	ABCB11	ns SNV	rs376255350	U/S	35	0.907	N/A
2	131043572	T	A	ARHGEF4	ns SNV	rs750383856	N/A	28.9	0.838	0.0005
2	169493760	T	C	BBS5	ns SNV	rs758508869	U/S	31	0.757	N/A
2	115689911	G	A	DPP10	ns SNV	rs200502140	N/A	31	0.785	0.0015
2	219238272	C	T	GLB1L	ns SNV	rs191524819	N/A	35	0.852	0.0005
2	127574021	A	C	MYO7B	ns SNV	rs559170047	N/A	26.7	0.763	0.0015
2	26463969	C	G	OTOF	ns SNV	rs199766465	N/A	32	0.85	0.007
2	165994164	C	T	SCN1A	ns SNV	rs121918808	Likely_benign	32	0.817	0.0025
2	166041286	A	G	SCN1A	ns SNV	rs773695263	CIP	25.9	0.884	N/A
2	85302590	T	C	TCF7L1	ns SNV	N/A	N/A	27.1	0.871	N/A

3	57106133	G	A	IL17RD	ns SNV	rs200088377	CIP	26.9	0.302	0.002
3	101245963	G	C	IMPG2	ns SNV	rs201905772	CIP	12.7	0.062	0.0075
3	130563212	A	G	COL6A6	ns SNV	rs527993234	N/A	23.1	0.889	0.0025
3	98580756	T	C	CPOX	ns SNV	rs143456081	Likely_benign	23.8	0.865	0.0035
3	58008647	A	T	FLNB	ns SNV	N/A	N/A	32	0.953	N/A
3	58154853	C	T	FLNB	ns SNV	rs369477886	N/A	34	0.876	N/A
3	52694955	A	G	GLT8D1	ns SNV	rs567530025	N/A	29.5	0.975	0.0005
3	193637229	A	G	OPA1	ns SNV	N/A	N/A	29.5	0.965	N/A
3	196947755	A	G	PIGZ	ns SNV	rs755420890	N/A	23.8	0.806	0.0005
3	30671923	A	T	TGFBR2	ns SNV	rs761231369	U/S	24.9	0.919	N/A
3	3137315	T	A	TRNT1	ns SNV	N/A	N/A	25.5	0.851	N/A
4	83439891	C	T	HELQ	ns SNV	rs758801143	N/A	34	0.831	0.002
4	108089142	T	C	LEF1	ns SNV	rs775583955	N/A	28.4	0.921	N/A
4	88046847	T	G	PKD2	ns SNV	rs772965121	N/A	25.9	0.838	0.0005
4	25124220	A	C	SEPSECS	ns SNV	N/A	N/A	27.2	0.935	N/A
4	6301275	G	A	WFS1	ns SNV	rs760692398	N/A	26.5	0.862	0.002
4	99057785	C	T	METAP1	ns SNV	N/A	N/A	26.3	0.835	N/A
5	90811089	C	T	ADGRV1	ns SNV	rs375450242	CIP	23.2	0.139	0.001
5	80654908	-	CAGCGCCCC	MSH3	nonframeshift insertion	rs747419370	CIP	N/A	N/A	N/A
5	16616886	G	A	RETREG1	ns SNV	rs528532732	CIP	11.63	0.027	0.0005
7	18027028	A	C	PRPS1L1	ns SNV	rs762277550	N/A	25.1	0.931	N/A
8	27463698	C	T	CHRN/A2	ns SNV	rs77710085	CIP	12.21	0.101	0.0025
8	142877162	G	C	CYP11B1	ns SNV	rs61751149	CIP	0.002	0.033	0.01
8	139731152	C	T	TRAPPC9	ns SNV	rs145503551	CIP	20.2	0.049	0.006
8	26652293	C	T	DPYSL2	ns SNV	rs779134278	N/A	35	0.773	N/A
8	24914676	C	A	NEFM	ns SNV	rs201666371	N/A	24.7	0.85	0.001
8	75556049	A	G	HNF4G	ns SNV	N/A	N/A	26.7	0.821	N/A
9	710876	A	G	KANK1	ns SNV	rs753436251	N/A	25.5	0.887	N/A
9	137463457	C	T	PNPLA7	ns SNV	rs140268990	N/A	31	0.89	0.0005
9	82990440	T	C	RASEF	ns SNV	N/A	N/A	27	0.909	N/A
10	60075299	G	A	ANK3	ns SNV	rs117475706	CIP	24.5	0.08	N/A
10	53857257	C	T	PCDH15	ns SNV	rs201137087	CIP	34	0.818	0.0065
10	93381386	C	T	MYOF	ns SNV	rs370493284	N/A	35	0.76	0.0005
10	3119925	C	T	PFKP	ns SNV	rs750120962	N/A	35	0.817	N/A
10	97042951	C	A	SLIT1	ns SNV	rs757038852	N/A	34	0.815	N/A

10	120905367	G	A	WDR11	ns SNV	rs201686484	N/A	34	0.792	0.003
10	31521176	T	C	ZEB1	ns SNV	rs746075441	N/A	24.7	0.867	N/A
10	53857257	C	T	PCDH15	ns SNV	rs201137087	CIP	34	0.818	0.0065
11	71435791	C	T	DHCR7	ns SNV	rs72954276	CIP	0.578	0.255	0.003
11	64808033	C	T	MEN1	ns SNV	rs607969	CIP	23.7	0.434	0.0045
11	794782	G	A	SLC25A22	ns SNV	rs142220309	CIP	23.6	0.51	N/A
11	6391623	-	C	SMPD1	frameshift insertion	rs756366019	Pathogenic	N/A	N/A	N/A
11	26537457	A	C	ANO3	ns SNV	N/A	N/A	27.3	0.871	N/A
11	93759821	C	T	C11orf54	ns SNV	rs181140249	N/A	34	0.862	0.004
11	94102955	G	A	HEPHL1	ns SNV	rs779789404	N/A	27.1	0.79	N/A
11	68448865	G	T	LRP5	ns SNV	rs147618989	U/S	24.1	0.842	0.0015
11	89400337	C	T	NOX4	ns SNV	rs184098470	N/A	33	0.867	0.0005
11	9847077	T	C	SBF2	ns SNV	rs754241592	N/A	32	0.945	0.002
12	21882797	C	T	ABCC9	ns SNV	rs141999048	CIP	21.4	0.371	0.0015
12	69586813	G	A	CCT2	ns SNV	rs772758306	N/A	32	0.942	0.0005
12	52490934	A	G	KRT6A	ns SNV	N/A	N/A	23.8	0.815	N/A
12	52428679	A	T	KRT75	ns SNV	rs192170292	N/A	27.3	0.908	0.0025
12	21642074	C	T	LDHB	ns SNV	rs200163319	N/A	24.5	0.844	0.005
12	103706942	T	G	STAB2	ns SNV	rs748243785	N/A	25.8	0.873	N/A
12	76815177	C	T	ZDHHC17	ns SNV	rs763263581	N/A	34	0.883	N/A
13	102875337	T	G	BIVM-ERC(	ns SNV	rs368550097	CIP	7.842	0.119	0.004
13	32338731	A	G	BRCA2	ns SNV	rs117187202	CIP	0.395	0.146	0.0005
13	32340191	T	C	BRCA2	ns SNV	rs80358811	CIP	0.002	0.14	0.0005
13	24906608	G	A	CENPJ	ns SNV	rs193181742	CIP	0.023	0.01	0.005
13	40799148	C	G	SLC25A15	ns SNV	rs187685447	CIP	13.64	0.355	0.0025
13	36879563	T	C	SMAD9	ns SNV	rs397514715	Pathogenic	24.8	0.902	0.0005
13	49551459	C	T	RCBTB1	ns SNV	N/A	N/A	35	0.981	N/A
13	36879563	T	C	SMAD9	ns SNV	rs397514715	Pathogenic	24.8	0.902	0.0005
14	77299507	G	C	POMT2	ns SNV	rs764015186	CIP	7.404	0.224	0.0025
14	24607627	G	T	GZMH	ns SNV	N/A	N/A	25.5	0.886	N/A
15	73367687	G	A	HCN4	ns SNV	rs201375192	CIP	20.5	0.271	0.0065
15	72346626	C	T	HEXA	ns SNV	rs575121167	CIP	32	0.711	0.0035
15	72353090	A	T	HEXA	ns SNV	rs185797496	Likely_benign	25.6	0.761	0.005
15	89320863	C	T	POLG	ns SNV	rs760305377	U/S	33	0.964	N/A
15	48139019	T	G	SLC24A5	ns SNV	rs192454382	Likely_benign	27.9	0.814	0.0045

16	23635054	C	A	PALB2	ns SNV	rs75023630	CIP	24.3	0.088	0.0075
16	72122957	G	A	PMFBP1	stopgain	rs140352254	Pathogenic	41	N/A	0.0005
16	3979202	G	A	ADCY9	ns SNV	N/A	N/A	34	0.805	N/A
16	55529843	C	T	LPCAT2	ns SNV	rs549291060	N/A	35	0.898	N/A
16	56870725	T	C	SLC12A3	ns SNV	rs749698843	N/A	27.9	0.814	N/A
16	984739	A	C	SOX8	ns SNV	N/A	N/A	24.9	0.865	N/A
17	80065503	G	A	CCDC40	ns SNV	rs185157579	CIP	7.049	0.051	0.0075
17	80108767	G	A	GAA	ns SNV	rs2229224	CIP	1.285	0.245	0.0035
17	58206158	C	T	MKS1	ns SNV	rs199910690	CIP	32	0.711	0.006
17	81935121	G	-	PYCR1	frameshift deletion	rs758601634	Pathogenic	N/A	N/A	N/A
17	50167954	C	T	SGCA	ns SNV	rs186669379	CIP	23.5	0.864	0.006
17	48623029	C	T	HOXB9	ns SNV	rs753182609	N/A	32	0.775	0.0045
17	21703291	G	C	KCNJ18	ns SNV	N/A	N/A	26.3	0.947	N/A
17	41480743	C	T	KRT35	ns SNV	rs201523783	N/A	24.3	0.902	0.0005
17	10511876	A	G	MYH1	ns SNV	rs201061123	N/A	22.2	0.882	0.0015
17	31232747	A	G	NF1	ns SNV	rs757222815	U/S	28.8	0.771	0.0005
17	39107433	C	T	PLXDC1	ns SNV	rs757389182	N/A	34	0.926	N/A
17	50167954	C	T	SGCA	ns SNV	rs186669379	CIP	23.5	0.864	0.006
17	9545244	T	A	STX8	ns SNV	rs201798916	N/A	28.7	0.796	0.003
18	31068905	G	A	DSC2	ns SNV	rs142410803	CIP	35	0.499	0.0085
19	38505076	G	A	RYR1	ns SNV	rs566495420	CIP	24.7	0.316	0.005
19	8090246	G	A	FBN3	ns SNV	rs183278638	N/A	34	0.798	0.001
19	3157825	T	C	GN/A15	ns SNV	N/A	N/A	23.3	0.861	N/A
19	48836366	C	T	HSD17B14	ns SNV	rs113246661	N/A	34	0.752	0.0035
20	3889380	C	G	PANK2	ns SNV	rs199680057	CIP	24.3	0.39	0.01
20	19886740	C	T	RIN2	ns SNV	rs183028833	CIP	16.18	0.008	0.0025
20	46725912	C	A	SLC2A10	ns SNV	rs777604168	CIP	23.7	0.625	0.002
20	34285586	C	T	AHCY	ns SNV	rs765418686	N/A	24.5	0.785	0.0005
20	20503382	G	A	RALGAPA2	ns SNV	rs374993331	N/A	29	0.865	0.002
20	17531195	C	A	BFSP1	ns SNV	N/A	N/A	28.4	0.775	0.0005
21	46416553	G	A	PCNT	ns SNV	rs201503338	CIP	8.188	0.011	0.001
22	26625533	C	T	CRYBA4	stopgain	rs199635152	Pathogenic	35	N/A	N/A
22	20973844	A	G	AIFM3	ns SNV	rs764765760	N/A	24.1	0.826	N/A
22	26628384	G	A	CRYBA4	ns SNV	N/A	N/A	31	0.873	N/A
22	29278078	G	T	EWSR1	ns SNV	N/A	N/A	29.4	0.781	N/A

X	32310266	C	T	DMD	ns SNV	rs148135406	CIP	17.97	0.147	0.0005
X	32441307	C	G	DMD	ns SNV	rs200213555	CIP	25.8	0.312	0.003
X	32644145	C	T	DMD	ns SNV	rs189143447	CIP	27.4	0.294	0.0035
X	153725702	T	A	ABCD1	ns SNV	rs782720024	Benign	25.8	0.915	0.002

Chr: chromosome; Ref: reference; Alt: alternative; ns SNV: non-synonymous single-nucleotide variant; CIP: Conflicting_interpretations_of_pathogenicity.
N/A: not applicable; U/S: Uncertain_significance; TWB AF: Allele frequency in Taiwan Biobank.
