# Supplementary material for: Identification of Genetic Variants Associated with Severe Myocardial Bridging through Whole-Exome Sequencing
Source: J Pers Med. 2023 Oct 18;13(10):1509. doi: 10.3390/jpm13101509 (PMC10608235; doi:10.3390/jpm13101509)
Supplement: Supplementary file 1 [file jpm-13-01509-s001.zip › Supplement S3_Detailed Functional Annotation of SMB candidate genes.pdf]

Supplement S3. Detailed Functional Annotation of SMB candidate genes.

Enriched gene set	Gene set size	Overlap genes	Gene Symbol	Gene Name
**Knockout mouse phenotype**				
Abnormal soleus morphology	21	4	Myof	myoferlin
			Ryr1	ryanodine receptor 1, skeletal muscle
			Sgca	sarcoglycan, alpha
			Ttn	titin
Impaired skeletal muscle contractility	38	6	Col6a3	collagen, type VI, alpha 3
			Dmd	dystrophin, muscular dystrophy
			Myh1	myosin, heavy polypeptide 1, skeletal muscle, adult
			Ryr1	ryanodine receptor 1, skeletal muscle
			Sgca	sarcoglycan, alpha
			Ttn	titin
Absent startle reflex	39	5	Adgrv1	adhesion G protein-coupled receptor V1
			Gpsm2	G-protein signalling modulator 2 (AGS3-like, C. elegans)
			Kcnj10	potassium inwardly-rectifying channel, subfamily J, member 10
			Myh1	myosin, heavy polypeptide 1, skeletal muscle, adult
			Pcdh15	protocadherin 15
Decreased skeletal muscle mass	107	8	Col6a3	collagen, type VI, alpha 3
			Dmd	dystrophin, muscular dystrophy
			Gaa	glucosidase, alpha, acid
			Myh1	myosin, heavy polypeptide 1, skeletal muscle, adult
			Nf1	neurofibromin 1
			Polg	polymerase (DNA directed), gamma
			Ryr1	ryanodine receptor 1, skeletal muscle
			Ttn	titin
Abnormal skeletal muscle mass	121	8	Col6a3	collagen, type VI, alpha 3
			Dmd	dystrophin, muscular dystrophy
			Gaa	glucosidase, alpha, acid
			Myh1	myosin, heavy polypeptide 1, skeletal muscle, adult
			Nf1	neurofibromin 1
			Polg	polymerase (DNA directed), gamma
			Ryr1	ryanodine receptor 1, skeletal muscle
			Ttn	titin
Abnormal muscle fiber morphology	322	13	Adgrv1	adhesion G protein-coupled receptor V1
			Cenpj	centromere protein J
			Col6a3	collagen, type VI, alpha 3
			Dmd	dystrophin, muscular dystrophy
			Gaa	glucosidase, alpha, acid
			Myof	myoferlin
			Opa1	OPA1, mitochondrial dynamin like GTPase
			Polg	polymerase (DNA directed), gamma
			Ryr1	ryanodine receptor 1, skeletal muscle
			Ryr2	ryanodine receptor 2, cardiac
			Sgca	sarcoglycan, alpha
			Trim63	tripartite motif-containing 63
			Ttn	titin
Increased or absent threshold for auditory brainstem response	310	12	Adgrv1	adhesion G protein-coupled receptor V1
			Dmd	dystrophin, muscular dystrophy
			Ewsr1	Ewing sarcoma breakpoint region 1
			Gpsm2	G-protein signalling modulator 2 (AGS3-like, C. elegans)

			Il17rd	interleukin 17 receptor D
			Kcnj10	potassium inwardly-rectifying channel, subfamily J, member 10
			Myh1	myosin, heavy polypeptide 1, skeletal muscle, adult
			Opa1	OPA1, mitochondrial dynamin like GTPase
			Otof	otoferlin
			Pcdh15	protocadherin 15
			Polg	polymerase (DNA directed), gamma
			Zeb1	zinc finger E-box binding homeobox 1
			Col6a3	collagen, type VI, alpha 3
			Dmd	dystrophin, muscular dystrophy
			Gaa	glucosidase, alpha, acid
			Myh1	myosin, heavy polypeptide 1, skeletal muscle, adult
			Myof	myoferlin
			Nf1	neurofibromin 1
Abnormal skeletal muscle morphology	381	14	Ntrk1	neurotrophic tyrosine kinase, receptor, type 1
			Polg	polymerase (DNA directed), gamma
			Ryr1	ryanodine receptor 1, skeletal muscle
			Sgca	sarcoglycan, alpha
			Trappc9	trafficking protein particle complex 9
			Trim63	tripartite motif-containing 63
			Ttn	titin
			Xdh	xanthine dehydrogenase
			Abcc9	ATP-binding cassette, sub-family C (CFTR/MRP), member 9
			Adcy9	adenylate cyclase 9
			Arhgef4	Rho guanine nucleotide exchange factor (GEF) 4
			Bbs5	Bardet-Biedl syndrome 5 (human)
			Cyp11b1	cytochrome P450, family 11, subfamily b, polypeptide 1
			Ddost	dolichyl-di-phosphooligosaccharide-protein glycotransferase
			Dmd	dystrophin, muscular dystrophy
			Dsc2	desmocollin 2
			Gaa	glucosidase, alpha, acid
			Hcn4	hyperpolarization-activated, cyclic nucleotide-gated K+ 4
			Lrp5	low density lipoprotein receptor-related protein 5
Abnormal cardiovascular system physiology	1421	29	Men1	multiple endocrine neoplasia 1
			Myof	myoferlin
			Nexn	nexilin
			Nf1	neurofibromin 1
			Nox4	NADPH oxidase 4
			Opa1	OPA1, mitochondrial dynamin like GTPase
			Pcnt	pericentrin (kendrin)
			Pkd2	polycystic kidney disease 2
			Psen2	presenilin 2
			Ryr2	ryanodine receptor 2, cardiac
			Scn1a	sodium channel, voltage-gated, type I, alpha
			Sgca	sarcoglycan, alpha
			Slc12a3	solute carrier family 12, member 3
			Tcf7l1	transcription factor 7 like 1 (T cell specific, HMG box)
			Tgfbr2	transforming growth factor, beta receptor II
			Trappc9	trafficking protein particle complex 9
			Ttn	titin

			Zeb1	zinc finger E-box binding homeobox 1
Abnormal cardiovascular system morphology	1794	34	Abcc9	ATP-binding cassette, sub-family C (CFTR/MRP), member 9
			Adcy9	adenylate cyclase 9
			Arhgef4	Rho guanine nucleotide exchange factor (GEF) 4
			Cenpj	centromere protein J
			Cyp11b1	cytochrome P450, family 11, subfamily b, polypeptide 1
			Dhcr7	7-dehydrocholesterol reductase
			Dmd	dystrophin, muscular dystrophy
			Flnb	filamin, beta
			Gaa	glucosidase, alpha, acid
			Il17rd	interleukin 17 receptor D
			Lef1	lymphoid enhancer binding factor 1
			Lrp5	low density lipoprotein receptor-related protein 5
			Men1	multiple endocrine neoplasia 1
			Mks1	Meckel syndrome, type 1
			Nexn	nexilin
			Nf1	neurofibromin 1
			Nox4	NADPH oxidase 4
			Opa1	OPA1, mitochondrial dynamin like GTPase
			Palb2	partner and localizer of BRCA2
			Pcnt	pericentrin (kendrin)
			Polg	polymerase (DNA directed), gamma
			Ryr1	ryanodine receptor 1, skeletal muscle
			Ryr2	ryanodine receptor 2, cardiac
			Sgca	sarcoglycan, alpha
			Slc2a10	solute carrier family 2 (facilitated glucose transporter), member 10
			Smad9	SMAD family member 9
			Tcf7l1	transcription factor 7 like 1 (T cell specific, HMG box)
			Tgfbr2	transforming growth factor, beta receptor II
			Trappc9	trafficking protein particle complex 9
			Trim63	tripartite motif-containing 63
			Ttn	titin
			Wdr11	WD repeat domain 11
			Zeb1	zinc finger E-box binding homeobox 1
**KEGG pathway**				
Arrhythmogenic right ventricular cardiomyopathy	72	7	ACTN2	actinin alpha 2
			DSC2	desmocollin 2
			DMD	dystrophin
			LEF1	lymphoid enhancer binding factor 1
			RYR2	ryanodine receptor 2
			SGCA	sarcoglycan alpha
			TCF7L1	transcription factor 7 like 1
**GO term categories**				
Detection of mechanical stimulus	43	6	ADGRV1	adhesion G protein-coupled receptor V1
			ANO3	anoctamin 3
			NTRK1	neurotrophic receptor tyrosine kinase 1
			PKD2	polycystin 2
			SCN1A	sodium voltage-gated channel alpha subunit 1
			TTN	titin
Muscle contraction	339	14	ACTN2	actinin alpha 2
			DMD	dystrophin
			DSC2	desmocollin 2
			ENO1	enolase 1
			GAA	glucosidase alpha, acid

			HCN4	hyperpolarization activated cyclic nucleotide gated potassium channel 4
			MYH1	myosin heavy chain 1
			MYOF	myoferlin
			RYR1	ryanodine receptor 1
			RYR2	ryanodine receptor 2
			SCN1A	sodium voltage-gated channel alpha subunit 1
			SGCA	sarcoglycan alpha
			TRIM63	tripartite motif containing 63
			TTN	titin
Muscle system process	423	14	ACTN2	actinin alpha 2
			DMD	dystrophin
			DSC2	desmocollin 2
			ENO1	enolase 1
			GAA	glucosidase alpha, acid
			HCN4	hyperpolarization activated cyclic nucleotide gated potassium channel 4
			MYH1	myosin heavy chain 1
			MYOF	myoferlin
			RYR1	ryanodine receptor 1
			RYR2	ryanodine receptor 2
			SCN1A	sodium voltage-gated channel alpha subunit 1
			SGCA	sarcoglycan alpha
			TRIM63	tripartite motif containing 63
			TTN	titin
Monovalent inorganic cation transport	513	15	ABCB11	ATP binding cassette subfamily B member 11
			ABCC9	ATP binding cassette subfamily C member 9
			ACTN2	actinin alpha 2
			ANK3	ankyrin 3
			DMD	dystrophin
			DPP10	dipeptidyl peptidase like 10
			HCN4	hyperpolarization activated cyclic nucleotide gated potassium channel 4
			KCNJ10	potassium voltage-gated channel subfamily J member 10
			KCNJ18	potassium voltage-gated channel subfamily J member 18
			PKD2	polycystin 2
			SCN1A	sodium voltage-gated channel alpha subunit 1
			SLC12A3	solute carrier family 12 member 3
			SLC24A5	solute carrier family 24 member 5
			SLC25A22	solute carrier family 25 member 22
			SLC2A10	solute carrier family 2 member 10
Inorganic cation transmembrane transport	722	19	ABCB11	ATP binding cassette subfamily B member 11
			ABCC9	ATP binding cassette subfamily C member 9
			ACTN2	actinin alpha 2
			ANK3	ankyrin 3
			DMD	dystrophin
			DPP10	dipeptidyl peptidase like 10
			HCN4	hyperpolarization activated cyclic nucleotide gated potassium channel 4
			KCNJ10	potassium voltage-gated channel subfamily J member 10
			KCNJ18	potassium voltage-gated channel subfamily J member 18
			OPA1	OPA1, mitochondrial dynamin like GTPase
			PKD2	polycystin 2
			RYR1	ryanodine receptor 1
			RYR2	ryanodine receptor 2

			SCN1A	sodium voltage-gated channel alpha subunit 1
			SLC12A3	solute carrier family 12 member 3
			SLC24A5	solute carrier family 24 member 5
			SLC25A22	solute carrier family 25 member 22
			SLC2A10	solute carrier family 2 member 10
			ZDHHC17	zinc finger DHHC-type containing 17
Cation transmembrane transport	810	20	ABCB11	ATP binding cassette subfamily B member 11
			ABCC9	ATP binding cassette subfamily C member 9
			ACTN2	actinin alpha 2
			ANK3	ankyrin 3
			DMD	dystrophin
			DPP10	dipeptidyl peptidase like 10
			HCN4	hyperpolarization activated cyclic nucleotide gated potassium channel 4
			KCNJ10	potassium voltage-gated channel subfamily J member 10
			KCNJ18	potassium voltage-gated channel subfamily J member 18
			OPA1	OPA1, mitochondrial dynamin like GTPase
			PKD2	polycystin 2
			RYR1	ryanodine receptor 1
			RYR2	ryanodine receptor 2
			SCN1A	sodium voltage-gated channel alpha subunit 1
			SLC12A3	solute carrier family 12 member 3
			SLC24A5	solute carrier family 24 member 5
			SLC25A15	solute carrier family 25 member 15
			SLC25A22	solute carrier family 25 member 22
			SLC2A10	solute carrier family 2 member 10
			ZDHHC17	zinc finger DHHC-type containing 17
Metal ion transport	841	20	ABCB11	ATP binding cassette subfamily B member 11
			ABCC9	ATP binding cassette subfamily C member 9
			ACTN2	actinin alpha 2
			ANK3	ankyrin 3
			DMD	dystrophin
			DPP10	dipeptidyl peptidase like 10
			HCN4	hyperpolarization activated cyclic nucleotide gated potassium channel 4
			HEPHL1	hephaestin like 1
			KCNJ10	potassium voltage-gated channel subfamily J member 10
			KCNJ18	potassium voltage-gated channel subfamily J member 18
			OPA1	OPA1, mitochondrial dynamin like GTPase
			PKD2	polycystin 2
			PSEN2	presenilin 2
			RYR1	ryanodine receptor 1
			RYR2	ryanodine receptor 2
			SCN1A	sodium voltage-gated channel alpha subunit 1
			SLC12A3	solute carrier family 12 member 3
			SLC24A5	solute carrier family 24 member 5
			WFS1	wolframin ER transmembrane glycoprotein
			ZDHHC17	zinc finger DHHC-type containing 17
Inorganic ion transmembrane transport	808	19	ABCB11	ATP binding cassette subfamily B member 11
			ABCC9	ATP binding cassette subfamily C member 9
			ACTN2	actinin alpha 2
			ANK3	ankyrin 3
			DMD	dystrophin
			DPP10	dipeptidyl peptidase like 10
			HCN4	hyperpolarization activated cyclic nucleotide gated potassium channel 4

			KCNJ10	potassium voltage-gated channel subfamily J member 10
			KCNJ18	potassium voltage-gated channel subfamily J member 18
			OPA1	OPA1, mitochondrial dynamin like GTPase
			PKD2	polycystin 2
			RYR1	ryanodine receptor 1
			RYR2	ryanodine receptor 2
			SCN1A	sodium voltage-gated channel alpha subunit 1
			SLC12A3	solute carrier family 12 member 3
			SLC24A5	solute carrier family 24 member 5
			SLC25A22	solute carrier family 25 member 22
			SLC2A10	solute carrier family 2 member 10
			ZDHHC17	zinc finger DHHC-type containing 17
Cation transport	1111	23	ABCB11	ATP binding cassette subfamily B member 11
			ABCC9	ATP binding cassette subfamily C member 9
			ACTN2	actinin alpha 2
			ANK3	ankyrin 3
			DMD	dystrophin
			DPP10	dipeptidyl peptidase like 10
			HCN4	hyperpolarization activated cyclic nucleotide gated potassium channel 4
			HEPHL1	hephaestin like 1
			KCNJ10	potassium voltage-gated channel subfamily J member 10
			KCNJ18	potassium voltage-gated channel subfamily J member 18
			OPA1	OPA1, mitochondrial dynamin like GTPase
			PKD2	polycystin 2
			PSEN2	presenilin 2
			RYR1	ryanodine receptor 1
			RYR2	ryanodine receptor 2
			SCN1A	sodium voltage-gated channel alpha subunit 1
			SLC12A3	solute carrier family 12 member 3
			SLC24A5	solute carrier family 24 member 5
			SLC25A22	solute carrier family 25 member 22
			SLC2A10	solute carrier family 2 member 10
			WFS1	wolframin ER transmembrane glycoprotein
			ZDHHC17	zinc finger DHHC-type containing 17
Ion transport	1608	29	ABCB11	ATP binding cassette subfamily B member 11
			ABCC9	ATP binding cassette subfamily C member 9
			ABCD1	ATP binding cassette subfamily D member 1
			ACTN2	actinin alpha 2
			ANK3	ankyrin 3
			ANO3	anoctamin 3
			CHRNA2	cholinergic receptor nicotinic alpha 2 subunit
			CYB5R1	cytochrome b5 reductase 1
			CYB5RL	cytochrome b5 reductase like
			DMD	dystrophin
			DPP10	dipeptidyl peptidase like 10
			HCN4	hyperpolarization activated cyclic nucleotide gated potassium channel 4
			HEPHL1	hephaestin like 1
			KCNJ10	potassium voltage-gated channel subfamily J member 10
			KCNJ18	potassium voltage-gated channel subfamily J member 18
			NF1	neurofibromin 1
			OPA1	OPA1, mitochondrial dynamin like GTPase

PKD2	polycystin 2
PSEN2	presenilin 2
RYR1	ryanodine receptor 1
RYR2	ryanodine receptor 2
SCN1A	sodium voltage-gated channel alpha subunit 1
SLC12A3	solute carrier family 12 member 3
SLC24A5	solute carrier family 24 member 5
SLC25A15	solute carrier family 25 member 15
SLC25A22	solute carrier family 25 member 22
SLC2A10	solute carrier family 2 member 10
WFS1	wolframin ER transmembrane glycoprotein
ZDHHC17	zinc finger DHHC-type containing 17

FDR, false discovery rate; KEGG, Kyoto Encyclopedia of Genes and Genomes; GO, Gene Ontology
